# Draft genome sequences of six high pH adapted *Marinobacter shengliensis* strains isolated from Mariana forearc serpentinite mud volcanoes

**DOI:** 10.1128/mra.01045-24

**Published:** 2024-12-20

**Authors:** Sabrina M. Elkassas, Margrethe H. Serres, Michael Navarro, Amina Patterson, Teodora Zhivkova, Madelyn Petersen, Katelyn Weeks, Susan Lang, Jeffrey Seewald, Geoffrey Wheat, Elizabeth Trembath-Reichert, Julie A. Huber

**Affiliations:** 1 Department of Marine Chemistry and Geochemistry, Woods Hole Oceanographic Institution, Woods Hole, Massachusetts, USA; 2 Department of Earth, Atmospheric, and Planetary Sciences, Massachusetts Institute of Technology, Cambridge, Massachusetts, USA; 3 Department of Environmental Science and Technology, Holyoke Community College, Holyoke, Massachusetts, USA; 4 Department of Geology and Geophysics, Woods Hole Oceanographic Institution, Woods Hole, Massachusetts, USA; 5 Department of Physics, Photonics and Optical Engineering, Bridgewater State University, Bridgewater, Massachusetts, USA; 6 School of the Earth, Ocean and Environment, University of South Carolina, Columbia, South Carolina, USA; 7 School of Molecular Sciences, Arizona State University, Tempe, Arizona, USA; 8 Global Undersea Research Unit, University of Alaska Fairbanks, Fairbanks, Alaska, USA; 9 School of Earth and Space Exploration, Arizona State University, Tempe, Arizona, USA; Rochester Institute of Technology, Rochester, New York, USA

**Keywords:** alkaliphile, serpentinite mud volcano, *Marinobacter*

## Abstract

Six marine bacterial isolates were obtained from fluid and sediments collected at alkaline serpentinite mud volcanoes of the Mariana forearc to examine life at high pH in a marine environment. Here, we present the draft genome sequences of these six isolates, classified as strains of the species *Marinobacter shengliensis*.

## ANNOUNCEMENT

Six strains (2B, PC6, EB4, 3B, 4B, and G5) of *Marinobacter shengliensis* were isolated from alkaline, serpentinized subseafloor fluid and sediment cores collected from three serpentinite mud volcanoes along the Mariana forearc (Western Pacific Ocean; Table 1). All cultures were isolated in MJYTGL medium at pH 10.5 (1, 2). Isolation was carried out using dilution-to-extinction methods described in (3). The NaHCO_3_/Na_2_CO_3_ buffer system was used for media pH 9 and above. Ranges of temperature, salinity, and pH that supported growth were determined under aerobic conditions on alkaline MJYTGL medium agar plates. Growth ranged from 0 to 13 wt% NaCl, pH 4.5–12 (optimum pH 9), and 15–35°C. These bacteria were chosen for sequencing due to their high pH growth conditions and metabolic versatility. Their genomes offer insights into microbial adaptations to high pH in deep marine systems.

**TABLE 1:** Metadata for the six *Marinobacter shengliensis* isolates

Parameter			Microbial isolates					
			2B	PC6	EB4	3B	4B	G5
*Environmental data*	*Geographic location*	*Region*	Mariana forearc, Southwest Pacific Ocean					
		*Site name*	Fantangisña (Celestial)			Asùt Tesoru (Big Blue)		Yinazao (Blue Moon)
		*IODP borehole*	U1497D			U1496C		U1492D
		*Geographic coordinates*	16°32.2548′N 147°13.2621′E			18°06.6068′N 147°06.1001′E		15°42.5694′N 147°10.5991′E
		*Depth (in meters below sea level*)	2019			1213		3666
		*Collection date*	Nov 2022			Dec 2022		
		*Biome*	Submarine serpentinite mud volcano					
		*Sample type*	Serpentinized sediment					Formation fluid
		*Sampling method*	Push core					Water sampler
		*pH*	7.7			12.4		7.4
		*Enrichment medium*	Alkaline, anaerobic MJYTGL (2)	Alkaline DSM282 (4)	Enceladus medium	Alkaline, anaerobic MJYTGL (2)	Alkaline, anaerobic MJYTGL (2)	Alkaline, anaerobic MJYTGL (2)
		*Isolation medium*	Alkaline, anaerobic MJYTGL (2)					
*Sequencing*		*DNA extraction kit*	MasterPure Complete DNA and RNA Purification Kit *LGC Biosearch Technologies* *Kit: Cat. # MC85200 and MC89010* *Reagents within the kit* *ProteinaseK @ 50 µg/µL (DNase- and RNase-free)(50% glycerol, 50 mM Tris-HCl [pH 7.5], 0.1 M NaCl, 0.1 mM EDTA, 1 mM DTT, 10 mM CaCl2, 0.1% Triton^®^ X-100): Cat. # MPRK092* *Tissue and Cell Lysis Solution: Cat. # MTC096H* *MPC Protein Precipitation Reagent: Cat. # MMP03750* *RNase A @ 5 µg/µL (50% glycerol, 25 mM NaOAc [pH 4.6]): Cat. # MRNA092* *TE buffer (10 mM Tris-HCl [pH 7.5], 1 mM EDTA): Cat. # MTE0970*					
		*DNA quantification instrument*	Qubit 4 Fluorometer					
		*Illumina library preparation*	Illumina DNA Prep, (M) tagmentation kit, and IDT For Illumina DNA/RNA Unique Dual Indexes Cat. # 20060060 and 20027213					
		*Sequencing technology*	llumina NextSeq2000, 300-cycle flow cell kit Cat. # 20100982					
		*No. of illumina reads (Mbp*)	292.7	321.7	255.1	298	301.7	213.5
		*Sequencing center*	SeqCoast Genomics, Portsmouth, NH					
		*Sequencing coverage*	69.71×	76.58×	56.51×	66.01×	67.10×	48.64×
		*Assembler*	SPAdes v3.15.3 (5)					
		*No. of contigs*	120	120	107	107	109	100
		*Largest contig (bp*)	389,944	389,944	324,043	324,043	300,860	325,043
		*N50 (bp*)	143,195	139,103	125,363	101,254	108,117	108,114
		*ORF caller*	Prodigal v2.6.3 (6)					
*Genomic features*		*Genome size (bp*)	4,198,766	4,200,595	4,514,035	4,514,217	4,496,379	4,389,321
		*Genome completeness (7*)	100%	100%	100%	100%	100%	100%
		*Genome contamination (7*)	0.43%	0.43%	0.43%	0.43%	0.43%	0
		*G + C content (mol%) (6*)	57.25	57.25	57.11	57.11	57.12	57.17
		*No. of protein-coding genes (6*)	3700	3697	3977	3976	3960	3860
		*No. of CRISPR loci (6*)	6	6	11	11	11	11
	*No. of RNA genes (8*)	*No. of 5S rRNA genes*	1	1	1	1	1	1
		*No. of 16S rRNA genes*	1	1	1	2	2	2
		*No. of 23S rRNA genes*	1	1	1	1	3	3
		*No. of tRNA genes*	45	45	45	47	45	44
	*Viral sequences (9*)	*Virsorter2 viral sequence categories*	2,3	2,3	1,2,3	2,3	2,3	2,3
		*Virsorter2 viral sequence types*	ssDNA	dsDNA, ssDNA	dsDNA, ssDNA	dsDNA, ssDNA	dsDNA, ssDNA	ssDNA
		*geNomad provirus contigs*	0	0	4	4	4	2
	*Average nucleotide identity genome comparisons (10*)	*2B*		100.0	96.12	96.17	96.20	96.16
		*PC6*	100.0		96.16	96.17	96.20	96.19
		*EB4*	96.18	96.17		100.0	100.0	99.99
		*3B*	96.14	96.16	99.98		100.0	100.0
		*4B*	96.16	96.10	99.99	99.99		99.95
		*G5*	96.14	96.17	99.99	100.0	99.99	
		*Marinobacter shengliensis* strain LZ6	95.86	95.85	96.65	96.66	96.65	96.66
		*Marinobacter shengliensis* strain SL013A3432	96.14	96.10	96.54	96.53	96.53	96.52
		*Marinobacter shengliensis* strain IOP 41	96.69	96.67	96.20	96.22	96.23	96.20
		*Marinobacter shengliensis* strain D49	96.45	96.45	96.99	96.97	97.03	96.96
		*Marinobacter alkaliphilus* strain JY28	96.21	96.20	96.60	96.64	96.66	96.61

For DNA extraction, microbial isolates were grown at 30°C in 7 mL of unreduced pH 10.5 MJYTGL liquid medium in Balch tubes with a headspace of 100% N_2_ (microaerophilic conditions) until visibly turbid. Genomic DNA was extracted using the MasterPure Complete DNA and RNA Purification Kit (LGC BioSearch Technologies) with added overnight DNA precipitation. Samples were prepared for whole genome sequencing using Illumina DNA Prep, (M) tagmentation, and IDT for Illumina DNA/RNA Unique Dual Indexes per manufacturer protocols. Sequencing was performed on the Illumina NextSeq2000 platform using a 300-cycle flow cell kit to produce 2 × 150 bp paired-end reads, with a sequencing coverage of 49–77X (Table 1).

**Fig 1:**
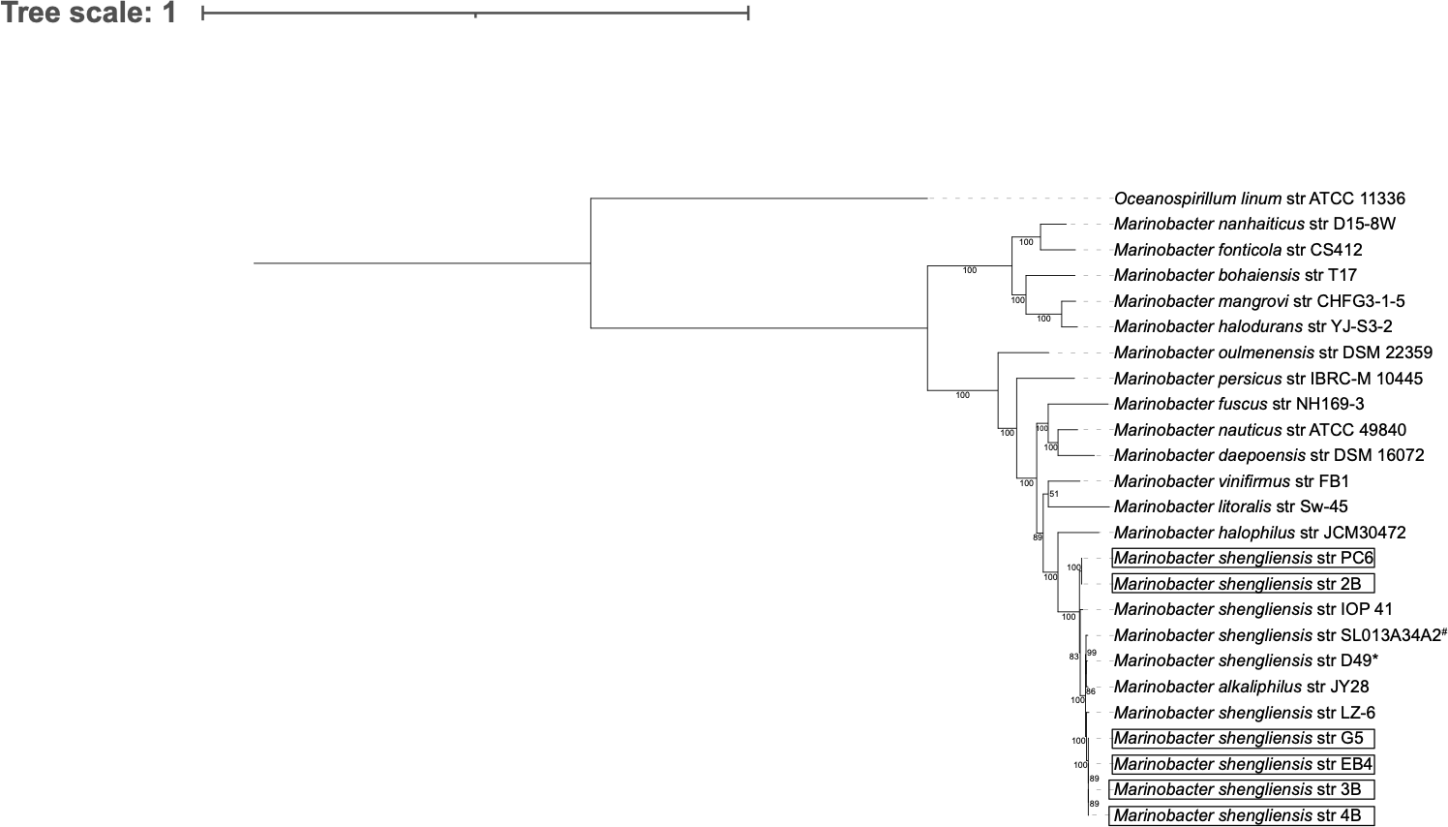
Neighbor-joining phylogenetic tree of all representative genomes of genus *Marinobacter* from GenBank and placement of isolates 2B, PC6, EB4, 3B, 4B, and G5 (black boxes) within species *Marinobacter shengliensis*. # denotes the type strain, *Marinobacter shengliensis* SL013A34A2, and * denotes the reference genome *Marinobacter shengliensis* str D49. *Oceanospirillum linum* str ATCC 11336 was included as an outgroup. The tree was generated using maximum-likelihood estimates based on alignments of nucleotide sequences within GToTree v1.8.4 (16) and IQTREE2 v2.3.0 (17). Bootstrap values >51% are shown at nodes (1000 replicates). The tree scale indicates the number of nucleotide substitutions per site.

Genome sequences were analyzed using the Department of Energy Systems Biology Knowledgebase (KBase) with default software parameters (11). Sequences were quality-checked with FastQC v0.12.1 (12), trimmed using Trimmomatic v0.36 (13), assembled using SPAdes v3.15.3 (5), and were found to be 100.0% complete, with 0–0.43% contamination using CheckM v1.0.18 (7). Quality-checked assemblies (Quast v4.4 [14]) resulted in genome sizes of 4,198,766–4,514,217 bp, maximum contig lengths of 300,860–389,944 bp, N_50_ values of 101,254–143,195 bp, and G + C content from 57.11% to 57.25%. Classification with GTDB-Tk v1.7.0 (15) revealed all genomes are *Marinobacter shengliensis* (16). *Marinobacter alkaliphilus* JY28, previously isolated from the Mariana forearc, clustered with other *Marinobacter shengliensis* strains, indicating all are the same species (2, 17, 18; Fig. 1). Average nucleotide identities, determined by FastANI v0.1.3, were >95%, further supporting this (10). Open reading frames predicted using Prodigal v2.6.3 (6) and annotated using DRAM v0.1.2 (19) and RASTtk v1.073 (8), resulted in 3839–4179 protein-coding genes, 1–3 complete rRNA genes, 44–47 tRNA genes, and 6–11 CRISPR loci among the six genomes. Virsorter2 v1.0.1 (9) detected viral sequences (scores > 0.9) in all genomes (Table 1).

All isolates contain genes for heterotrophic growth under varying oxygen levels, including cytochrome c oxidases, dissimilatory nitrate reductase, nitrite reductase, D-lactate dehydrogenase, and degradation of aromatic compounds and peptide bonds. Genes encoding enzymes for CO_2_ assimilation, such as pyruvate synthase, suggest that the isolates may perform heterotrophic CO_2_ assimilation when nutrient conditions are limited (20). The ability of the isolates to grow at pH 12 may be linked to Na^+^:H^+^ antiporters for maintaining intracellular homeostasis in alkaline surroundings (21).

## Data Availability

Raw sequence reads for the genomes of all six *Marinobacter shengliensis* are available at NCBI under BioProject accession no. PRJNA1154273, and BioSample and SRA accession nos. SAMN43409841, SRR30477260, and SRR30491032 (isolate 2B), SAMN43409842, SRR30477259, and SRR30491031 (isolate PC6), SAMN43409843, SRR30477258, and SRR30491030 (isolate EB4), SAMN43409844, SRR30477257, and SRR30491029 (isolate 3B), SAMN43409845, SRR30477256, and SRR30491028 (isolate 4B), SAMN43409846, SRR30477255, and SRR30491027 (isolate G5). Assembled and annotated contigs are available under GenBank accession nos. JBHFLB000000000 (isolate 2B), JBHFLC000000000 (isolate PC6), JBHFLD000000000 (isolate EB4), JBHFLE000000000 (isolate 3B), JBHFLF000000000 (isolate 4B), and JBHFLG000000000 (isolate G5).
